# Evaluation of the Accuracy of Electrocardiogram Interpretation in Emergency and Critical Care Medicine Residents in Addis Ababa, Ethiopia: A Cross-Sectional Study

**DOI:** 10.1155/emmi/6711086

**Published:** 2025-09-28

**Authors:** Meron Tesfaye Akalu, Demmelash Gezahegn Nigatu, Tseganesh Mekonnen Hailemariam, Mikiyas Gifawosen Teferi

**Affiliations:** ^1^Department of Emergency and Critical Care, School of Medicine, College of Health Sciences, Addis Ababa University, Addis Ababa, Ethiopia; ^2^School of Medicine, College of Health Sciences, Addis Ababa University, Addis Ababa, Ethiopia

**Keywords:** accuracy, critical care residents, electrocardiogram interpretation, emergency

## Abstract

**Background:** An electrocardiogram is a diagrammatic representation of a heart's electrical activity. This technique can detect life-threatening conditions within minutes. It is one of the major investigative modalities at which emergency physicians should be accurate. The accuracy of emergency residents varies from country to country, with improvement in interpretation as the year of residency increases. There have been no published papers on ECG interpretation among emergency residents until now, but studies on graduating medical students have shown low competency.

**Methods:** A cross-sectional study was conducted on emergency and critical care medicine residents of Tikur Anbessa Specialized Hospital and Saint Paul Millennium Medical College. Data were collected from April 2021 to September 2021 via a structured questionnaire. The data were entered, cleaned, edited, and analyzed via SPSS Version 26.0 statistical analysis software. Descriptive statistics and bivariate and multivariate binary logistic regressions were used to analyze the data.

**Results:** Fifty-seven emergency and critical care medicine residents were able to participate in this study, 33 (57.9%) of whom were from Addis Ababa University and 24 (42.1%) of whom were from Saint Paul Millennium Medical College. The average percentage of EMCC residents who interpreted the ECGs was 29.5%. Only 10 residents (17%) were able to correctly interpret > 50% of the ECGs. Most of the residents who participated in this study were Year 1 residents, followed by Year 2 residents. Among the 15 ECG abnormalities, the commonly identified were polymorphic ventricular tachycardia, normal sinus rhythm, and a double-chamber pacemaker. The year of residency (AOR 3.34) was found to be significantly associated with greater performance in ECG interpretation.

**Conclusion:** According to this study, the interpretation accuracy of ECGs by emergency medicine and critical care residents is low, which is comparable to the findings of studies performed in South Africa and Australia.

## 1. Introduction

The electrocardiogram is a diagrammatic representation of the heart's electrical activity, which is one of the lifesaving investigative modalities that should be available at the emergency department (ED) [1]. ECG findings might be the only information available at hand to diagnose life-threatening rhythm abnormalities, electrolyte imbalances, toxicological abnormalities, and myocardial infarction [2]. The other significant use of ECG is identifying the arrest rhythm in cardiac arrest patients and leading physicians to the subsequent management of the patient [3]. Since it is easily available, noninvasive, and can be interpreted within minutes, it has immeasurable value in saving lives in the ED, which demands that emergency physicians be experts in interpreting the findings and applying them to clinical use [4].

Emergency and critical care medicine specialty is limited by a lack of investigative modalities and the difficulty of disposing of patients [5]. Thus, in this resource-limited setting, the availability of ECGs and their proper use for proper emergency patient management are invaluable. Although no studies have been conducted in Ethiopia on ECCM resident competencies and the accuracy of interpretation of ECG findings, previous studies conducted in Australia and South Africa have shown that the level of accuracy increases as seniority increases [2, 4].

Ethiopia is a developing country, and ECCM is one of the evolving specialty trainings currently being given only in four teaching hospitals throughout the country. During most working hours, both consultants and residents are available at the ED, but during duty hours, residents are the most senior physicians available. Although there are no papers published on the common ED presentation of patients at Tikur Anbessa Specialized Hospital (TASH), according to the research conducted in Turkey in 2019, cardiovascular complaints are among the most common presentations [6]. Among the common ECG findings of acute myocardial ischemia, both ST-segment and T-wave abnormalities are misinterpreted, according to the Journal of Internal Medicine published in 1992 [7]. ECG is only as good as the physician who interprets it. The correct interpretation of ECG abnormalities is one of the crucial skills that every ECCM resident should be able to acquire. Inability to do so can negatively affect patient outcomes and might also lead to fatal outcomes.

Until now, there have been no published papers in Ethiopia regarding the evaluation of the competencies of emergency medicine residents in ECG interpretation. However, studies evaluating the competence of ECG interpretation in graduating medical students have shown low competency [3].

An evaluation of the accuracy of ECG interpretation among ECCM residents will help provide insights into the available gaps in the prioritization of patients. This study will also have a significant impact on patient disposition and prevent ED overcrowding.

This study provides more information on the accuracy with which emergency medicine residents interpret the common ECG abnormalities they face in the ED. The study also recommends that department heads improve residents' ECG interpretation skills. The main purpose of this study is to assess emergency resident accuracy in ECG interpretation in two medical colleges, identify the gaps, and identify the causes of the gaps.

## 2. Methods and Materials

### 2.1. Study Setting

The study will be conducted in Addis Ababa at AAU (TASH) and SPMMC. AAU was established in 1950, and the School of Medicine was founded in 1972. According to the official website of the university, the college currently offers eight undergraduate programs and over 70 postgraduate programs. The TASH is the teaching hospital of the college. The TASH is the largest hospital in Ethiopia, with over 700 beds. The Department of Emergency Medicine was formed at the TASH in collaboration with AAU, the University of Wisconsin, and the University of Toronto. The department launched a three-year residency program and a two-year EMCC nursing program after being approved by the Senate of AAU in October 2010. The first four emergency and critical care specialists graduated in October 2013 [8].

St. Paul's Millennium Medical College was formed in 2010. It is governed by a board under the Federal Ministry of Health [9]. According to the college website, the college has more than 2800 clinical, academic, and support staff with an average inpatient capacity of 700 beds. The college sees an average of 1200 emergency and outpatient clients daily. Saint Paul University established an ED in 2011 [9].

### 2.2. Study Design

A cross-sectional study of emergency medicine residents was conducted in 2021 from July 2021 to August 2021.

### 2.3. Source Population

The source population in this study was emergency and critical care medicine residents.

### 2.4. Study Population

Emergency and critical care medicine residents at the AAU and SPMMC were available at the time of the study.

### 2.5. Sample Size

The sample size formula for the cross-sectional study design is given by the single population proportion formula denoted by

(1) $$\begin{array}{c} n=\frac{{\left(Z\left(\alpha /2\right)\right)}^{2}\;p\left(1-p\right)}{d^{2}}. \end{array}$$

Here, *n* is the minimum required sample size, *Zα*/2 is the value under the standard normal table for a given confidence interval (CI) (1.96 for 95% CI), *p* is the best estimate of prevalence since we do not have a previous study in our country, 50% is used to increase the strength of the study, and *d* is the margin of error (0.05).

(2) $$\begin{array}{c} n=\frac{{\left(1.96\right)}^{2}0.5\left(1-0.5\right)}{0.05^{2}}=384. \end{array}$$

Since our source population is 100, we used the correction formula where n is the sample size we calculated (384), and *N* is our total population (100),

(3) $$\begin{array}{c} \text{Corrected sample size}=\frac{n\times N}{n+N}\approx 80. \end{array}$$

Adding 10% nonresponse, the sample size calculated is 88, since there is no significant difference between the calculated sample size and the study population. All ECCM residents who are currently on training at AAU and SPMMC and those who are available and at the time of the study were enrolled.

### 2.6. Inclusion and Exclusion Criteria

#### 2.6.1. Inclusion Criteria

All the emergency and critical care medicine residents attended their residency program at the SPMMC and TASH were included.

#### 2.6.2. Exclusion Criteria

Residents who were not willing or unable to participate were excluded.

### 2.7. Variables

Dependent variable: accuracy in ECG interpretation includes the following.

Independent variables are given as follows.

• Year of residency.
• ECG interpretation lectures.
• Self-teaching.
• Availability of ECG machines.
• Asking for help.

### 2.8. Operational Definition

Accuracy, which is 80%, is defined from a previous GMS study on ECG interpretation.

Performance: The total score of the participants out of the 15 ECGs is changed into a percentage, and higher performance (score) is scored as correctly interpreting > 50% of the ECGs, and lower performance (score) is scored as incorrectly interpreting > 50% of the ECGs (which is 8 ECGs and above).

Senior residents: Residents who are in the 3rd year of their study and Year 2 residents who completed the first 6 months of their Year 2.

Junior residents: Residents in their first year of residency and Year 2 residents before the first 6 months of Year 2.

ECG training—either online or in-person training on ECG interpretation.

ECG interpretation: Correctly interpreting a given EC.

### 2.9. Data Collection Tool and Procedure

ECGs were selected from previous South African studies, ECG web blogs, and standard ECG textbooks, and the questionnaires from the previous studies were modified and structured. The questionnaires included basic information about the residents and ECGs, with a focus on common life-threatening events that occur in the ED. A cardiologist, an emergency medicine consultant, and interpretations from blogs and books were used to standardize the ECG interpretation, and two similar answers were used as the correct answers to the ECG reading. Thus, on the basis of these criteria, out of the 20 selected ECGs, 15 of the images fulfilled the criteria and were presented to the residents for reading. The primary investigator was the data collector, and the completeness of the data was checked before the completion of data collection.

### 2.10. Data Processing

The completed questionnaire was coded, manually checked, entered into Excel, and exported to SPSS Version 26 for cleaning and analysis by the principal investigator.

Descriptive statistics, proportions, means, and medians were calculated via tables and charts to characterize the study population on the basis of sociodemographic and background characteristics. The first association between each independent variable and the dependent variable was assessed via bivariate analysis. Then, those independent variables with *p* values < 0.2 were subjected to multivariate logistic regression to control for confounders and to identify predictors of competency in ECG interpretation. A Hosmer–Lemeshow goodness-of-fit test was used to check the model's fitness, and a *p* value of > 0.05 was taken as the fit. In the multivariate logistic regression, a *p* value of < 0.05 was used as a criterion for a statistically significant association. An adjusted odds ratio with a 95% CI was calculated to determine the presence and strength of the association.

### 2.11. Ethical Considerations

A formal letter was taken from Addis Ababa University College of Health Sciences, Department of Emergency and Critical Care Medicine, to obtain approval to conduct this study. Participation was voluntary after written informed consent was obtained. The privacy of the participants was maintained by not writing their names in the questionnaire.

### 2.12. Dissemination of Results

**Figure 1:**
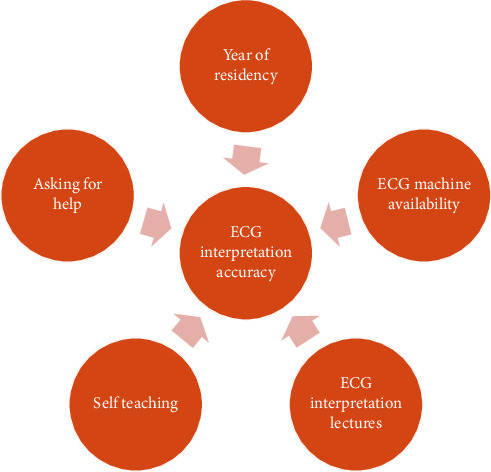
Relationships between the various independent variables and the outcome variable.

The findings of this study will be disseminated to Addis Ababa University College of Health Science and Saint Paul Millennium Medical College. It will be presented at symposiums and conferences. The publication of the study will be considered at the end (Figure 1).

## 3. Results

### 3.1. Returns

The nonresponse rate in this study was 28.75%. At the time of the study, 98 eligible participants were included, 80 questionnaires were distributed, and only 57 of them were returned to the primary investigator (71.25%).

### 3.2. Characteristics of the Study Participants

**Figure 2:**
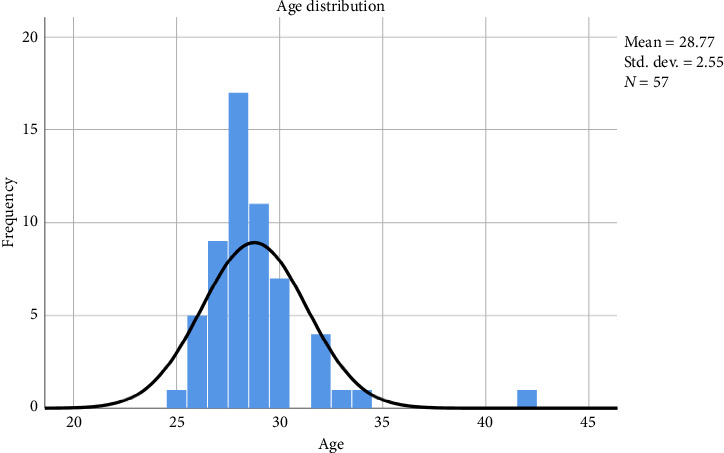
Age distribution of the participants.

In this study, 57 emergency and critical care medicine residents (male: *n* = 42, 73.7%; female: *n* = 15, 23.3%), with a mean age of 28.77 ± 2.77 (*R* = 25–42) years from both AAU (57.4%) and SPMMC (42.6%), participated and completed the questionnaires, as shown in Figure 2.

Most of the residents who participated in this study were Year 1 residents (*n* = 28; 49.1%), followed by Year 2 residents (*n* = 18; 31.6%), and finally, Year 3 residents (*n* = 11; 19.3%).

**Figure 3:**
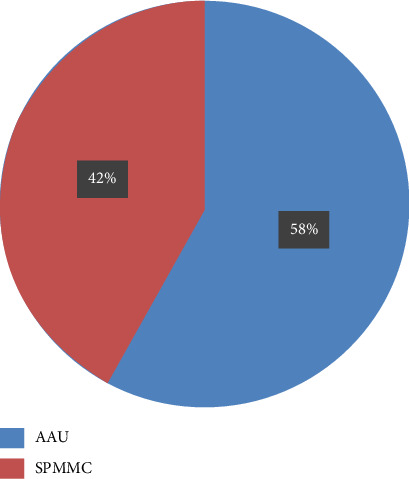
Place of postgraduate specialty training.

Thirty-three participants (57.9%) were from AAU, whereas the remaining 24 (42.1%) were from SPMMC, as shown in Figure 3.

Twenty-four (42.1%) of the residents had worked as general practitioners for 1-2 years, 21 (36.8%) had worked for more than 2 years, and the remaining 12 (21%) had worked for less than one year.

All the residents had ECG classes in the first year of their residency, of which 49 (87.5%) attended all the classes. Among the 57 residents, 45 (80.4%) said that the ECG class was not enough.

Twenty-nine (50.9%) of the residents felt that, on average, 25%–50% of ED patients require ECG as a diagnostic tool per day.

**Figure 4:**
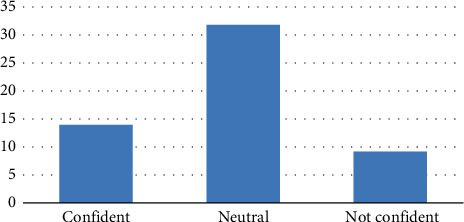
Participants' confidence ratings.

Most of the residents, 32 (58.2%), rated their confidence level of ECG interpretation as neutral, and 14 (25.5%) rated it as confident, as shown in Figure 4.

Forty-seven (82.5%) of the residents usually asked for help during ECG interpretation, and senior residents were mentioned as a source of help for 40 (78.4%) of the participants.

All of the study participants said that nothing bad had ever happened because of their ECG interpretation.

Most of the residents, 35 (61.4%), said that they update their ECG knowledge whenever they have an assignment and a case, and the sources for most of them were textbooks and online lectures (82.5% and 56.1%, respectively).

Thirty-four (60.7%) of the residents labeled their satisfaction with the current ECG training as fair; the others equally labeled their satisfaction as poor and good.

The majority of the residents recommended frequent lectures, case-based discussion, and available ECG machines to improve the current teaching method.

Most of the residents (*n* = 2; 49.1%) worked their general practitioner year at a primary hospital, and the majority of them claimed (*n* = 35; 61.4%) that ECG machines were not available at the time of their practice.

### 3.3. ECG Findings' Interpretation

In general, no resident scored 80% (12 and above) in the interpretation of the ECGs. Thus, the accuracy of emergency and critical care medicine residents in interpreting ECGs was found to be poor. The highest score was 11 (73.3%), the lowest score was 0, and the average score was 4.42 (29.4%), as shown in Table 1.

**Figure 5:**
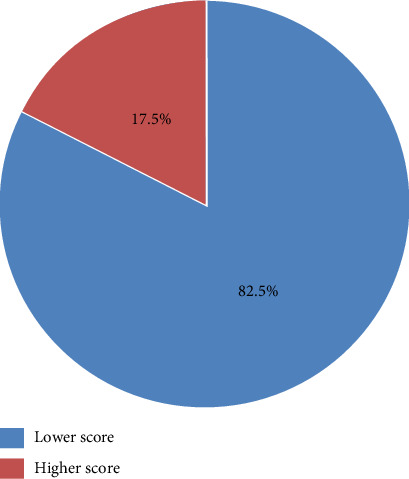
High and low scores of the participants.

Ten residents had scores > 50% (8 and above), and the remaining 47 residents had scores < 50% (7 and below), as shown in Figure 5.

In this study, of 57 participants, only 5 (8.8%) were able to identify pathological Q waves in the inferior leads.

Thirty-two of the participants (56.1%) were incorrect in identifying inferolateral MI.

Sixteen (28.6%) of the participants were able to identify Wellens syndrome correctly.

Forty-three of the participants (75.4%) incorrectly interpreted pacemaker failure (failure to capture) in a paced rhythm.

Thirteen of the study participants (22.8%) were correct in interpreting prolonged QT intervals.

Among the 57 participants, 20 (35.7%) were correct in identifying LBBB.

Fifteen (26.8%) of the participants correctly identified the Mobitz Type 2 AV block. In addition, 32 (57.1%) cases were incorrect. Forty-eight (87.3%) of the participants were incorrect in accurately identifying WPW with the AFIB, and only 5 (9.1%) of the participants were correct.

Twenty-six (46.4%) of the participants correctly identified the pacing rhythm.

Only 5 (8.8%) participants correctly identified ECG findings of pericarditis, and 41 (71.9%) of the participants were incorrect. Twenty-four (43.6%) of the participants correctly identified pulsus alternans and low-voltage rhythms.

Only 9 (16.4%) of the participants identified inferior wall MI with RV infarction, and 39 of the participants (70.9%) were incorrect. Twenty-eight (50%) of the participants were able to correctly identify normal sinus rhythm. Twenty-six (45.6%) of the participants were able to identify RBBB. Thirty-seven (64.9%) of the participants identified polymorphic ventricular tachycardia (V TACH) correctly, as shown in Table 2.

**Figure 6:**
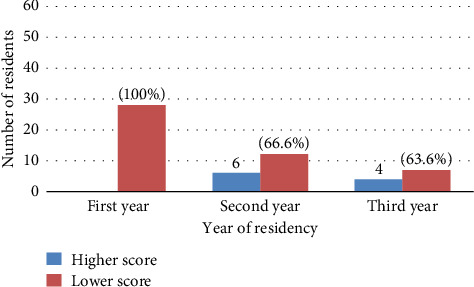
Years of residency and performance level.

When we observe participants' scores and year of residency, all of the first-year residents scored below 50%, which is defined as a low score, whereas 66.6% and 63.6% of the second- and third-year residents, respectively, scored low scores (Figure 6).

### 3.4. Associated Factors

Bivariate logistic regression was performed to assess the association of each independent variable with the outcome variable (high or low score), and the three variables that had *p* values < 0.2 were year of residency, updating oneself on ECG interpretations, and asking for help during ECG interpretation.

In the multivariate logistic regression, an increase in the year of residency was significantly associated with greater performance in interpreting ECGs after controlling for other variables (AOR = 3.34 [1.1, 10.2]) at a *p* value < 0.05 (Table 3).

## 4. Discussion

The American College of Physicians, in their article about training and competency evaluation for the interpretation of 12 lead electrocardiograms, proposed that ECG interpretation is a skill that requires an understanding of the general disease's condition, which results in an ECG abnormality and correlates the patient with the ECG abnormality. They also suggested that although interpretation errors are significant among cardiologists and noncardiologists (4% to 33%), fatality from these errors is approximately 1% [10, 11].

According to a prospective cross-sectional double-blinded study that was performed in Australia at Victoria University on emergency medicine residents to assess whether ECG interpretation accuracy improves with increasing years of emergency medicine training, which enrolled 122 trainees in total, 48 (39%) were senior trainees, 74 (60.6%) were other trainees, and the overall accuracy of ECG interpretation was 67.5% (95% CI 63.2%–71.8%) for the senior trainees and 49.6% (95% CI 45.2%–53.9%) for the other trainees. This study concluded that ECG interpretation improves in accuracy with increasing years of training [2].

In a study that was performed in the United States by the Virginia University Department of Emergency Medicine to assess electrocardiogram interpretation, training, and competency in the residency program, which used an interactive survey instrument posted on the Internet via software and emailed to program directors (PDS), which used 125 officially recognized councils for graduate medical education–approved U.S. EM residency programs, 99 respondents (response rate 79.2%) completed the online survey. Instructions (99%), case-based lectures (98%), and educational lectures (98%) were the most common methods for teaching interpretations of ECGs, followed by computer-based instructions (34%) and ECG laboratories (12%). Finally, most of the PDSs were comfortable with the ability of residents to interpret ECGs 96% by the third year and 91% by the fourth year of residency. Finally, the PDS of EM concluded that the EM residency program is sufficient for preparing graduates for ECG interpretation [12].

In a study performed in New York, Albert Einstein University College of Medicine, to assess the competency and accurate interpretation of ECGS among internal medicine and emergency residents, a study that included 120 participants, 87 internal medicine residents, and 33 emergency residents took a test interpreting 12 lead ECGSs and recorded their diagnosis and certainty, after which 2 cardiologists established the correct diagnosis. The results of the study revealed that there was no significant difference in overall competency between emergency medicine and internal medicine residents. They finally concluded that even though there was an improvement in clinical experience, overall competency was low [13].

A prospective cross-sectional study in South Africa was conducted with 55 emergency residents, 49 of whom completed the assessment (49%), and the average ECG interpretation score was 46.4% (95% CI 41.5%–51.2%). The improvement in ECG interpretation increased as seniority increased. The junior group had an overall average score of 42.2% (95% CI 36.9%–47.5%), whereas the senior group had an average score of 52.2% (95% CI 43.4%–61.5%). Overall, this study concluded that the average score of 46.4% obtained in the study was lower than the score obtained from international countries where emergency medicine is a well-established subspecialty [4].

A cross-sectional study of graduating medical students at Addis Ababa University and Haramaya University, which included 202 participants, revealed that 61.3% (95% CI 56.3%–66.3%) of the participants were capable of correctly interpreting primary ECG parameters such as rates, rhythms, and axes. The arrest rhythms, asystole, pulseless electrical activity (PEA), V TACH, and ventricular fibrillation (V FIB) were recognized by 32.75% on average (95% CI 28.25%–37.25%). Only 19.3% were able to correctly interpret common life-threatening emergency conditions, including myocardial infarction, electrolyte abnormalities, and AV-BLOCKS.

This study concluded that graduating medical students have low competence in ECG interpretation [3]. Overall, the interpretations of the ECGs of no participants in this study were accurate (Score ≥ 80%). Even though there is no specific number of ECGs that need to be interpreted to define competency in emergency physicians, the American Heart Association recommends that the median number of ECGs that need to be interpreted by a general internist should be 100 and that a cardiologist should be 750 to define competency [14].

A previous study on graduating medical students at Addis Ababa and Haramaya University revealed that the ECG interpretation competency of graduating medical students was insufficient [3]. This study revealed that only 29.8% of the study participants and 16.4% of the participants experienced ST-elevation myocardial infarction of the inferolateral wall and ST-elevation myocardial infarction of the inferior wall with RV infarction, respectively. In a previous study that compared the competency of ECG interpretation among cardiologists and emergency physicians, especially in determining ST-segment elevation myocardial infarction, the results revealed that they have comparable skills, indicating that emergency physicians have a high rate of accuracy in interpreting ST-elevation myocardial infarctions in patients with chest pain [3].

This poor baseline suggests that residency programs must not only teach advanced ECG interpretation but also remediate foundational knowledge gaps left unaddressed in undergraduate training. Enhancing ECG education in medical schools should thus be prioritized in parallel with residency-level reforms.

In this study, the average score of ECG interpretation was 4.42 (29.4%) out of 15 ECGs (95% CI 0%–73.3%). A study that was performed in South Africa and published in 2010 reported that the overall average of ECG interpretation was 46.4% (95% CI 41.5%–51.2%) [2]. A study that was performed in Australia and published in 2007 to assess emergency physicians' accuracy in the interpretation of ECGs also revealed that the average accuracy score was 56.6% [2]. A study that was also published in the United States in 2005 to assess competency in ECG interpretation among emergency medicine residents and internal medicine residents reported that the average score of ECG interpretation was 60%, and the study also revealed that there was no significant difference in ECG interpretation between emergency residents and internal medicine residents [13].

Compared with other studies, our study revealed significantly lower performance in ECG interpretation. Several factors may explain this discrepancy. First, the extent and frequency of ECG-focused education may be more limited in Ethiopia. In our study, 80.4% of residents stated that ECG classes were insufficient. Second, ECG machines were often unavailable during prior general practice experience, reducing residents' exposure to real-world practice. Third, although the ECGs used in this study were standardized and reviewed by specialists, their difficulty might have exceeded what was used in other countries' assessments, limiting direct comparability.

This study revealed that there was a statistically significant association between residency and performance level (*p* value 0.05), which is comparable to the findings of studies conducted in South Africa, Australia, and the United States [2, 4, 13], which revealed that there was a significant improvement in identifying ECG abnormalities as the year of residency increased. This likely reflects a combination of increased clinical exposure and cumulative informal learning. However, all residents received only introductory ECG training in their first year, with limited structured reinforcement in subsequent years. Participants reported that didactics such as lectures, case-based discussion, and online videos were helpful, but irregular and often self-initiated. This differs from the programs in the United States and Australia, where ECG training is systematically distributed throughout residency years.

Improving ECG competency requires systematic curricular enhancements. While current teaching methods rely heavily on didactic lectures, expanding hands-on learning such as ECG interpretation labs, bedside teaching, and high-fidelity simulations could significantly enhance retention and diagnostic accuracy. Furthermore, integrating ECG-focused rotations, such as in cardiology wards or ICUs, may strengthen both skills and confidence.

The limitations of this study are that the sample size was small, and it did not include other ECCM residency program places in Ethiopia. The sampling technique was a convenience sampling method, which is predisposed to selection bias.

## 5. Conclusion

This study demonstrates that the ECG interpretation accuracy among ECCM residents in Addis Ababa is suboptimal, with a notable correlation between seniority and performance. Key contributing factors include inadequate didactic reinforcement, limited access to ECG machines, and weak preresidency ECG foundations. To address this gap, structured and continuous ECG education—through frequent case-based lectures, dedicated cardiology rotations, simulation labs, and improved undergraduate training—should be implemented across all residency years.

## Figures and Tables

**Table 1:** ECG interpretation scores of ECCM residents.

Score	Frequency	Percent
0	5	8.8
1	5	8.8
2	6	10.5
3	7	12.3
4	7	12.3
5	7	12.3
6	6	10.5
7	4	7
8	5	8.8
9	4	7
11	1	1.8
Total	57	100

**Table 2:** ECG findings and interpretation by participants.

ECG findings	Interpretation	
	Correct	Incorrect
Pathological Q wave	5 (8.8%)	52 (81.2%)
Inferolateral MI	17 (29.8%)	40 (70.3%)
Wellens syndrome	16 (28.6%)	41 (71.3%)
Pacemaker failure	6 (10.5%)	51 (89.5%)
Prolonged QT interval	13 (22.8%)	44 (81.2%)
LBBB	20 (35.1%)	37 (64.9%)
Mobitz Type 2 AV block	15 (26.8%)	42 (73.2%)
WPW with AFIB	5 (8.8%)	52 (91.2%)
Double chamber pacemaker	26 (45.6%)	31 (54.4%)
Pericarditis	5 (8.8%)	52 (91.2%)
Pulsus alternans	24 (42.1%)	33 (57.9%)
Inferior wall MI with RV infarction	9 (16.1%)	48 (83.9%)
Normal sinus rhythm	28 (49.1%)	29 (50.9%)
RBBB	26 (45.6%)	31 (54.4%)
Polymorphic V TACH	37 (64.9%)	20 (35.1%)

**Table 3:** Factors associated with better performance in ECG interpretation.

Variable	Bivariate logistic regression		Multivariate logistic regression	
	COR (95% CI)	*p* value	AOR (95% CI)	*p* value
Increment in the year of residency	4.3 (1.56, 11.87)	0.005	3.34 (1.1, 10.2)	0.03^∗^
Asking for help	0.16 (0.03, 0.8)	0.03	0.2 (0.03, 1.43)	0.1
Frequent updating oneself on ECG	0.36 (0.01, 1.2)	0.1	0.36 (0.01, 1.34)	0.13

*Note:* COR, reported crude odds ratios.

Abbreviations: AOR, adjusted odds ratio; CI, confidence interval.

^∗^Statistically significant variable.

## Data Availability

The datasets generated and/or analyzed during the current study are available from the corresponding author upon reasonable request.

## Nomenclature

AAU: Addis Ababa University
AV: Atrioventricular
ECG: Electrocardiography
ED: Emergency department
ECCM: Emergency and critical care medicine
PEA: Pulseless electrical activity
SPMMC: Saint Paul Millennium Medical College
STEMI: ST elevation myocardial infarction
TASH: Tikur Anbessa Specialized Hospital
LBBB: Left bundle branch block
RBBB: Right bundle branch block
WPW: Wolf Parkinson white
V TACH: Ventricular tachycardia
V FIB: Ventricular fibrillation
